# The *Drosophila* Prosecretory Transcription Factor *dimmed* Is Dynamically Regulated in Adult Enteroendocrine Cells and Protects Against Gram-Negative Infection

**DOI:** 10.1534/g3.115.019117

**Published:** 2015-05-20

**Authors:** Katherine Beebe, Dongkook Park, Paul H. Taghert, Craig A. Micchelli

**Affiliations:** * Department of Developmental Biology, Washington University School of Medicine, St. Louis, Missouri 63110; † Department of Anatomy and Neurobiology, Washington University School of Medicine, St. Louis, Missouri 63110

**Keywords:** *Drosophila*, midgut, immunity, enteroendocrine, *dimmed*

## Abstract

The endocrine system employs peptide hormone signals to translate environmental changes into physiological responses. The diffuse endocrine system embedded in the gastrointestinal barrier epithelium is one of the largest and most diverse endocrine tissues. Furthermore, it is the only endocrine tissue in direct physical contact with the microbial environment of the gut lumen. However, it remains unclear how this sensory epithelium responds to specific pathogenic challenges in a dynamic and regulated manner. We demonstrate that the enteroendocrine cells of the adult *Drosophila melanogaster* midgut display a transient, sensitive, and systemic induction of the prosecretory factor *dimmed* (*dimm*) in response to the Gram-negative pathogen *Pseudomonas entomophila (Pe)*. In enteroendocrine cells, *dimm* controls the levels of the targets *Phm*, *dcat-4*, and the peptide hormone, Allatostatin A. Finally, we identify *dimm* as a host factor that protects against *Pe* infection and controls the expression of antimicrobial peptides. We propose that *dimm* provides “gain” in enteroendocrine output during the adaptive response to episodic pathogen exposure.

The endocrine system mediates long-range peptide hormone signaling to broadcast environmental changes to target tissues via the circulatory system. Endocrine cells must therefore function as biological sensors that detect physiochemical stimuli and translate them into changes in peptide and amine signals. The diffuse enteroendocrine system of the gastrointestinal (GI) tract is notable for both its size and the diversity of its secretory products (Ahlman and Nilsson 2001; Rehfeld 2004). Embedded within the barrier epithelium of the GI tract, enteroendocrine cells are situated uniquely with respect to the complex microbial communities of the gut lumen. Secreted enteroendocrine peptide hormones regulate local processes such as peristalsis and intestinal secretion, as well as long-range effects on metabolism, immune response, and the nervous system (Nässel and Winther 2010; Helander and Fandriks 2012). Thus, enteroendocrine cells coordinate essential aspects of physiological homeostasis at the barrier epithelium.

Studies in mammals have demonstrated the ability of enteroendocrine cells to respond to bacterial challenge with secretion of peptides and changes in gene expression (reviewed in Furness *et al.* 2013). However, these studies were largely performed *in vitro* and focused on isolated enteroendocrine cell types. Precisely how the diffuse endocrine system responds to episodic challenge under physiological conditions and the molecular mechanisms that coordinate this adaptive endocrine response remain unknown.

The adult *Drosophila* gut is a useful model to investigate the function and regulation of the diffuse endocrine system. The population of endocrine cells can be readily detected along the entire anterior-posterior axis of the GI tract by the pan-enteroendocrine marker Prospero (Micchelli and Perrimon 2006; Ohlstein and Spradling 2006). The *Drosophila* midgut, like its mammalian counterpart, also expresses a diverse array of secretory peptide hormones that exhibit regional and local diversity along the GI tract (Veenstra 2009; Veenstra and Ida 2014; Beehler-Evans and Micchelli 2015). Many of these peptides are functionally conserved across species (Nässel and Winther 2010; Veenstra 2011). Importantly, the effects of microbes on the gut epithelium can also be studied in *Drosophila*. For example, *Pseudomonas entomophila* (*Pe*), a pathogenic Gram-negative bacteria isolated from the GI tract of wild *Drosophila*, is a potent stimulus promoting stem cell mediated regeneration of the adult midgut epithelium (Vodovar *et al.* 2005; Buchon *et al.* 2009; Jiang *et al.* 2009; Strand and Micchelli 2011). Although *Pe* has been shown to induce gene expression changes in some epithelial cell types, a response of the enteroendocrine cell population has not yet been described.

*dimm* encodes a NeuroD-related basic helix-loop-helix transcription factor that is expressed in a subpopulation of cells in the central and peripheral nervous system during development (Hewes *et al.* 2003). In *dimm* mutants, the levels of secretory neuropeptides in the central nervous system are diminished. Misexpression studies show that Dimm functions as a “master” regulator capable of conferring two essential properties of the regulated secretory pathway to cells that do not ordinarily display them; the ability to produce large dense core vesicles that store peptides and the enzymatic machinery necessary to posttranslationally process pro-forms into biologically active signals (Hamanaka *et al.* 2010). Consistent with this phenotype, genome-wide characterization of Dimm binding sites has led to the identification of a number of potential mediators of this process (Hadzic *et al.* 2015).

Here, we examine the adult *Drosophila* diffuse endocrine system and characterize the regulation and function of the prosecretory transcription factor *dimm* in response to pathogenic bacteria.

## Materials and Methods

### Fly stocks and culturing

The following stocks were used: Wild-type: *w^1118^* and *Canton-S*; *w; pros-LacZ, ry / TM3 ry, Sb, Ser*; *w, UAS-dcr2; NPFGal4 / CyO; tubGal80^TS^, UAS-GFP / TM6C*; *w; esgGal4, UAS-GFP, tubGal80^TS^ (esg^TS^)*; *w, UAS-dcr2; tubGal4 / CyO; UAS-GFP, tubGal80^TS^ / TM6C* (*tub^TS^*); *w, UAS-rpr, UAS-hid*; *y w; P[EPgy2] dimm^EY14636^* (BL#21432); *y w; dimm^Rev4^ / CyO* (*Rev4*, see Hewes *et al.* 2003); *y v*; *P[TRiP.JF02093]attP2* (BL#26976, designated “dimm^RNAi^”); *P[KK112513]VIE-260B* (BL#v104028, designated “Phm^RNAi^”). For additional information, see FlyBase (http:flybase.org).

**Figure 1:**
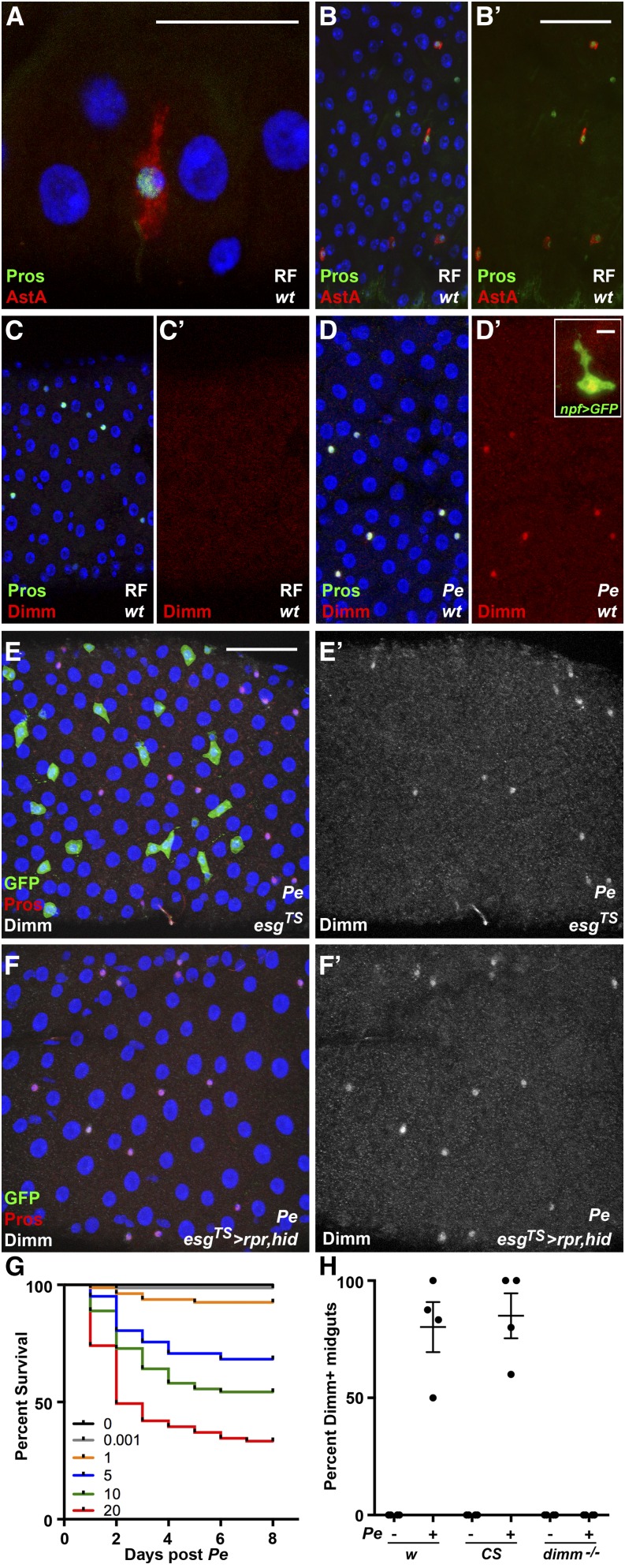
Adult enteroendocrine cells induce Dimm in response to the bacterial pathogen *Pseudomonas entomophila* (*Pe*). Gastrointestinal epithelium is shown under baseline and infected conditions. (A, B′) Confocal micrographs of the adult midgut epithelium stained for *pros*-LacZ and the peptide Allatostatin A (AstA) (DAPI, blue; anti-AstA, red; anti-βgal, green). (C, D′) Confocal micrographs of the adult midgut epithelium stained for Prospero (Pros) and Dimm after regular food (RF) (C, C′) or treatment with *Pe* (D, D′), (DAPI, blue; anti-Dimm, red; anti-Pros, green). Inset: representative cell from a *Pe* exposed midgut expressing *npf* > GFP (anti-Dimm, red; anti-GFP, green). (E, F′) Confocal micrographs of adult midguts temperature shifted to drive expression of GFP (E, E′) or the proapoptotic genes *rpr* and *hid* (F,F’) in *esg^TS^* expressing progenitor cells and subsequently exposed to *Pe* (DAPI, blue; anti-Pros, red; anti-GFP, green; anti-Dimm, white). Temperature shift to drive transgene expression was performed 3 d before exposure to *Pe*. RF controls are displayed in Figure S2. Scale bars: 10 µm (A), 50 µm (B, E), and 5 µm (D, inset). (G) Survival of wild-type females after a 24-hr exposure to a range of *Pe* doses (n = 4 trials, 80 females). (H) Quantification of the percentage of Dimm^+^ midguts per infection trial, comparing control genotypes and *dimm* mutants, *Pe* OD 5, 24 hr. Lines indicate mean values ± SEM.

Wild-type flies of the genotype *w^1118^ / Canton-S* were analyzed in all wild-type data with the exception of Figure 1H, where *w^1118^* and *Canton-S* were analyzed individually. The strong hypomorphic genotype *dimm^Rev4^* / *dimm^EY14636^* was analyzed in all *dimm* loss of function experiments.

Mated female flies were analyzed in all experiments. Females were collected the day of eclosion and aged 5 to 7 d before mock or *Pe* treatment. Females were 3 d of age in Supporting Information, Figure S1, C−E, where no mock or *Pe* treatments were administered. Flies were maintained on standard food media [*i.e.*, regular food (RF); Lee and Micchelli 2013] and supplemented with yeast paste every 2−3 d in all RF conditions. Flies were cultured at 25° and transferred to 29° at the time of mock or *Pe* treatment and time points thereafter.

### Antisera

#### Primary antibodies:

Primary antibodies included mouse anti-Allatostatin A (AstA) (1:20, DSHB); guinea pig anti-dCat-4 (CG13248) (1:500), (Park *et al.* 2011); guinea pig anti-Dimm (1:250), (Allan *et al.* 2005); chicken anti-GFP (1:10,000, Abcam); rabbit anti-Phm (1:750), (Kolhekar *et al.* 1997); mouse anti-Pros (1:100, DSHB); mouse anti-βGal (1:100, DSHB); and rabbit anti-βGal (1:2,000, Cappel).

#### Secondary antibodies:

Secondary antibodies used were goat anti-chicken Alexa Fluor 488 (1:2,000; Molecular Probes); goat anti-mouse Alexa Fluor 488 (1:2,000; Molecular Probes); goat anti-mouse Alexa Fluor 568 (1:2,000; Molecular Probes); goat anti-rabbit Alexa Fluor 568 (1:2,000; Molecular Probes); and goat anti-guinea pig Alexa Fluor 568 (1:2,000; Molecular Probes).

### Histology

Isolation and whole-mount immunostaining of adult midguts was performed as previously described in detail (Micchelli 2014). In summary, adult female flies were dissected in 1× phosphate-buffered saline (PBS; Sigma-Aldrich). The midgut was removed and fixed in 0.5× PBS, 4% electron microscopy-grade formaldehyde (Polysciences) overnight at 4°. Samples were washed in 1× PBS, 0.1% Triton X-100 (PBST) for a minimum of 2 hr, and then incubated in primary antisera overnight. Samples were subsequently washed for 2 hr in PBST, incubated in secondary antisera for 3 hr, and washed a minimum of 2 hr in PBST. DAPI containing Vectashield mounting media (Vector Laboratories) was used for mounting.

### Bacterial infection

Flies were infected *ad libitum* with *Pseudomonas entomophila* (*Pe*). Infected flies were fed on Tegosept-free food supplemented with 0.5mL of *Pe. Pe* bacterial culture was concentrated by centrifugation and resuspended in 5% sucrose. OD_600_ was measured and the concentrated *Pe* was subsequently diluted to the designated experimental OD. Mock-infected flies were placed on Tegosept-free food supplemented with 0.5 mL of 5% sucrose. Flies were shifted to and maintained at 29° throughout the course of the experiment after mock or *Pe* treatment. Exposure to mock or *Pe* food was 24 hr. Flies were returned to RF after treatment. The number of *Pe* colony-forming units per 0.5 mL of applied volume was estimated by counting the colony-forming units from a serial dilution of OD 20 resuspended *Pe* culture.

In lethality assays, adult female flies were collected in the first 24 hr of eclosion and aged 5−7 d. Flies were cultured at 25° at the ratio of 3 males + 20 females per vial. Survival of males was not included in the analysis. Flies were transferred to mock or *Pe* laced food of the designated OD and cultured at 29°. Flies were transferred to RF supplemented with yeast paste after 24h of *Pe* exposure. Lethality was assayed daily for 8 d.

### Imaging acquisition and quantification

Confocal images were collected using a Leica TCS SP5 microscope. Photoshop CS (Adobe) and ImageJ (National Institutes of Health) were used to process images for brightness and contrast. To analyze the percent of Dimm^+^ midguts, samples were classified as positive when they displayed >2 anti-Dimm^+^ cells in the superficial side of the entire anterior-posterior length. Dimm^+^, Pros^+^ values were obtained by analyzing projected maximum micrographs collected at 40× magnification along the entire anterior-posterior axis of each gut analyzed. Regional boundaries were defined by morphologic constrictions present in the medial portion. ImageJ software was used to measure mean intensity values. Specifically, using ImageJ, we defined particle areas using the anti-Pros channel and then measured mean intensity of the anti-Dimm channel in that area. Dimm levels were normalized for each micrograph. Levels were normalized using the following equation (R − B) / B, where R = mean intensity in the particle area and B = mean intensity of a midgut region outside particle areas. A similar method was used to collect AstA levels (Figure 4P), with the exception that particle areas were defined manually by tracing the AstA^+^ cytoplasmic area.

### Quantitative polymerase chain reaction (qPCR) analysis

Tissue was collected from whole bodies (n = 10 females per treatment per trial) and midguts (n = 20 females per treatment per trial) and was collected and analyzed from two to three infection trials. Head, leg, and wing tissue was excluded from whole body collection. Crop and malpighian tubule tissue was excluded from midgut collection. Total RNA was extracted using the RNeasy Mini Kit (QIAGEN) according to the manufacturer’s instructions. On-column DNase digestion with RNase-Free DNase set (QIAGEN) was performed to remove genomic DNA contamination. The quality and quantity of RNA were assessed using a NanoDrop 1000 Spectrophotometer (Thermo Scientific). cDNA was synthesized using the High-Capacity cDNA Reverse Transcription Kit according to the manufacturer’s protocol (Applied Biosystems). cDNA was subsequently treated with RNase H (New England Biolabs) for 30 min at 37°. qPCR was performed using an Applied Biosystems StepOnePlus System and SYBR Green PCR Master Mix (Applied Biosystems). The 2^−ΔΔ^*^C^*_T_ method was used to analyze fold change between mock and *Pe* treated samples (Livak and Schmittgen 2001). *RpL32* was used as a calibrator and verified in our experiments as a reliable standard unaffected by *Pe* treatment (Figure S3).

### Primers

Primer sequences are listed in Table S1. The *dimm* primers generated in this study were designed with the consideration that they span an exon−exon junction and were validated by serial dilution of cDNA and tested for melt curve specificity.

### Statistical analyses and sample sizes

Graphs and statistical analyses were generated using Prism GraphPad Software. Samples sizes below are listed as n = number of trials, number of samples analyzed in total per treatment.

#### For survival analysis:

Log-rank (Mantel-Cox) test was performed. Figure 1G and Table S2: n = 4 trials, 80 females. Figure 5A and Table S3: n = 4 trials, 80 females.

#### For percent expression analysis:

**Figure 2:**
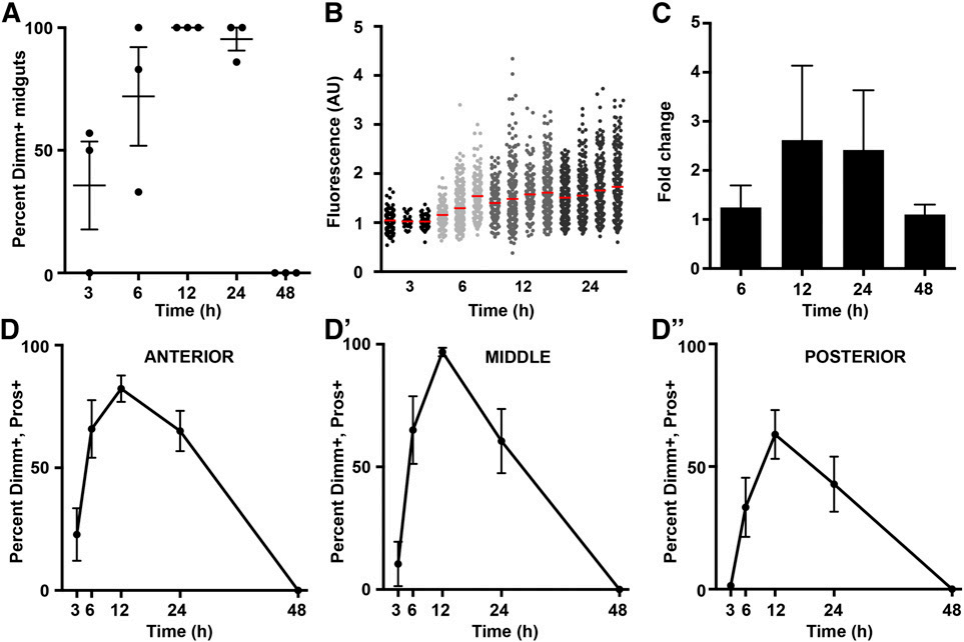
Dimm is transiently induced after *Pseudomonas entomophila* (*Pe*). Time course of *dimm* induction. (A) Quantification of the percentage of individuals in a given trial that displayed at least two positive anti-Dimm enteroendocrine cells over time after *Pe* (n = 3 trials, 18 midguts). (B) Quantification of fluorescence of anti-Dimm staining in individual Pros^+^ cells over time. Each column represents enteroendocrine cells from an individual midgut (n = 3−4 midguts, 35−345 enteroendocrine cells per midgut). Arbitrary units (AU). (C) Quantitative polymerase chain reaction analysis of *dimm* mRNA expression in midgut tissue over time (n = 2 trials, 40 midguts). Fold change represents *Pe* compared with mock using the 2^−ΔΔ^*^C^*_T_ method. (D-D′′) Regional analysis of the percentage of Dimm^+^ enteroendocrine cells per midgut over time (n = 3 trials, 18 midguts). *Pe* dose was OD 5 and time of collection as indicated. Mean values ± SEM are plotted.

**Figure 3:**
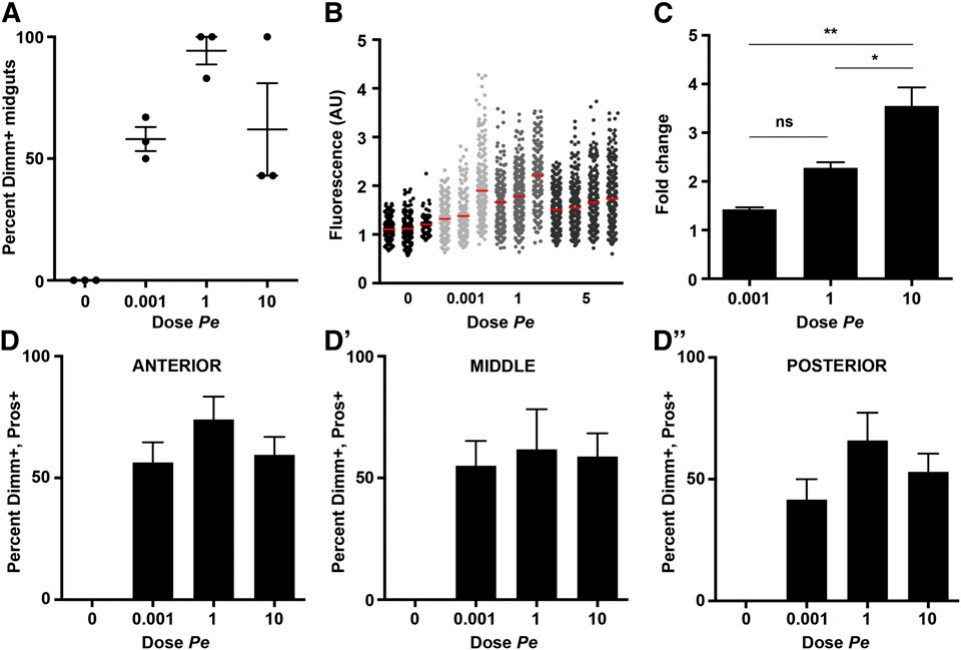
Dimm induction is sensitive to the dose of *Pseudomonas entomophila* (*Pe*). Dose response of *dimm* induction. (A) Quantification of the percentage of individuals in a given trial that displayed at least two positive anti-Dimm enteroendocrine cells over varying doses of *Pe* (n = 3 trials, 18 midguts). (B) Quantification of fluorescence of anti-Dimm staining in individual Pros^+^ cells over *Pe* dose. Each column represents enteroendocrine cells from an individual midgut (n = 3−4 midguts, 70−330 enteroendocrine cells per midgut). (C) Quantitative polymerase chain reaction analysis of *dimm* mRNA expression in midgut tissue over *Pe* dose (n = 3 trials, 60 midguts). Fold change represents *Pe* compared with mock using the 2^−ΔΔ^*^C^*_T_ method. (D-D′′) Regional analysis of the percentage of Dimm^+^ enteroendocrine cells per midgut over *Pe* dose (n = 3 trials, 18 midguts). Time of collection was 24 hr after *Pe* and dose as indicated. Mean values ± SEM are plotted.

**Figure 4:**
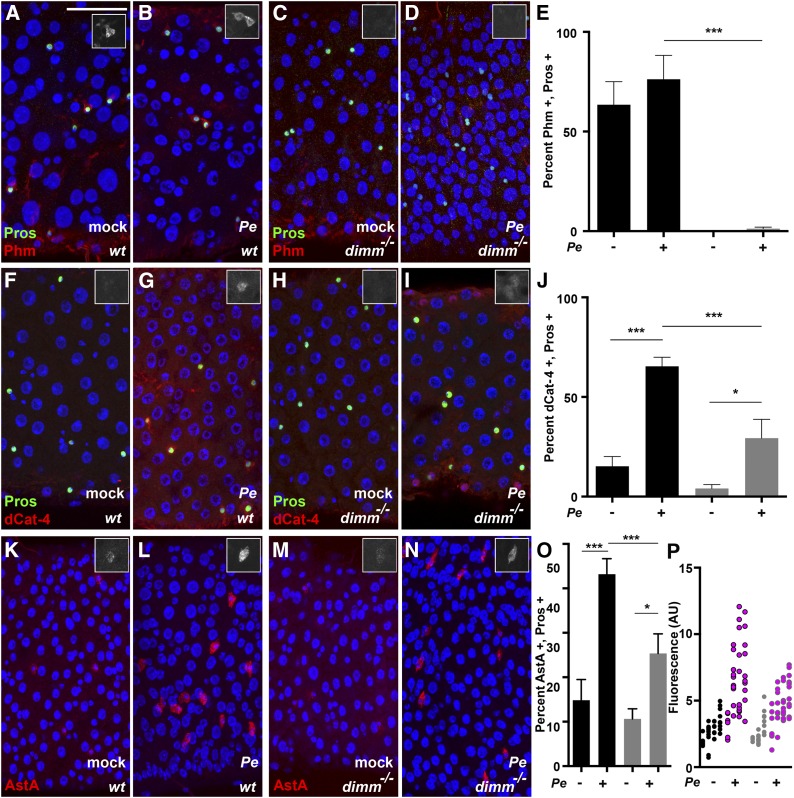
*dimm* target gene expression and peptide regulation in enteroendocrine cells. Analysis of *dimm* target genes. Insets show representative cells in gray scale to best permit comparison between samples. (A−E) Phm staining in wild-type and *dimm* mutant midguts after mock or *Pe* treatment. (DAPI, blue; anti-Phm, red; anti-Pros, green). (F−J) dCat-4 staining in wild-type and *dimm* mutant midguts after mock or *Pe* treatment. (DAPI, blue; anti-dCat-4, red; anti-Pros, green). (K−P) Allatostatin A (AstA) staining in wild-type and *dimm* mutant midguts after mock or *Pe* treatment. (DAPI, blue; anti-AstA, red). (E, J, and O) Percent positive cells of indicated antibody. Black bars, wild-type; gray bars, *dimm* mutants. Bars indicate mean values ± SEM (n = 2−3 trials, 12−18 midguts). (P) Quantification of mean fluorescence of anti-AstA staining in the AstA^+^ cell population after mock or *Pe* treatment. Wild-type mock, black; wild-type *Pe*, pink with black border; *dimm* mutant mock, gray; *dimm* mutant *Pe*, pink with gray border, (n = 1 trial, 3−4 midguts, 3−11 AstA^+^ cells per midgut). *Pe* dose was OD 5 and time of collection 24 hr in all experiments. Scale bar: 50 µm.

One-way analysis of variance (ANOVA) was performed, followed by Tukey’s multiple comparisons. Percent Dimm^+^ midguts and Dimm^+^ cells (Figure 1, Figure 2, and Figure 3): n = 3−4 trials, 18−24 midguts. Percent Phm^+^ cells (Figure 4E): n = 3 trials, 18 midguts. Percent dCat-4^+^ cells (Figure 4J): n = 2 trials, 12 midguts. Percent AstA^+^ cells (Figure 4O): n = 3 trials, 18 midguts.

#### For fluorescence intensity:

Dimm (Figure 2B and Figure 3B): n = 1 trial, 3−4 midguts, 35−345 enteroendocrine cells. AstA (Figure 4P): n = 1 trial, 3−4 midguts, 3−11 enteroendocrine cells.

#### For qPCR analysis:

**Figure 5:**
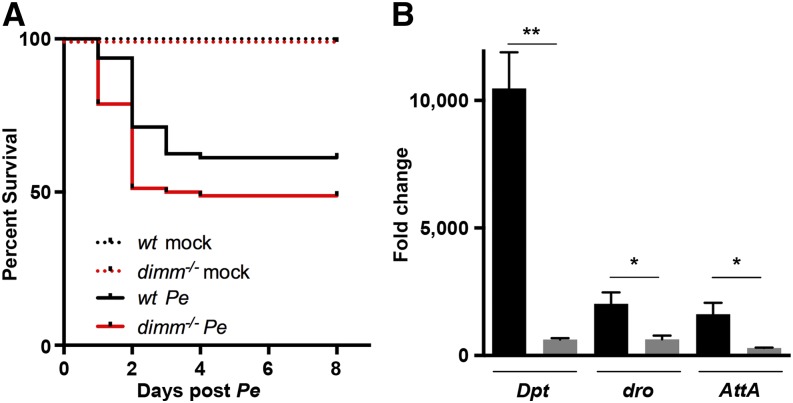
*dimm* is a host factor that protects against *Pseudomonas entomophila* (*Pe*) infection. Survival and immune response of *dimm* mutants after Gram-negative *Pe* infection. (A) Survival of wild-type and *dimm* mutants after exposure to mock or *Pe* treatment. Flies were exposed to *Pe* OD 10 for 24 hr (n = 4 trials, 80 females). (B) Quantitative polymerase chain reaction analysis of mRNA in whole-body tissue of a subset of antimicrobial peptides in wild-type and *dimm* mutant genotypes, *Pe* OD 5, collected 24 hr after *Pe* (wild-type, black bars; *dimm* mutant, gray bars). Fold change represents *Pe* compared with mock using the 2^−ΔΔ^*^C^*_T_ method (n = 3 trials, 30 flies). Bars indicate mean values ± SEM.

One-way ANOVA (Figure 2C and Figure 3C) and unpaired *t*-test (Figures 5B and Figure S1) were performed. *dimm* expression (Figure 2C): n = 2 trials, 40 midguts, (Figure 3C): n = 3 trials, 60 midguts, (Figure S1): n = 3 trials, 30 whole bodies. AMP expression (Figures 5B and Figure S3): n = 3 trials, 30 whole bodies. *upd3* expression (Figure S3): n = 3 trials, 60 midguts. The qPCR data presented in Figure 3C was analyzed from samples that met a quality control criterion for *Pe* infectivity. Specifically, a parallel survival assay group displayed significant lethality at 48h following *Pe* OD 20 exposure.

#### For mutant analysis:

For mutant analysis (Figure S1), an unpaired *t*-test was performed. Body mass: n = 3 trials, 70-90 females. Midgut area: n = 2 trials, 14−19 midguts. Prospero cell density: n = 2 trials, 16 midguts.

## Results

### Mature enteroendocrine cells induce Dimm in response to *Pe* infection

We hypothesized that cells of the diffuse endocrine system would stain positively for Dimm protein, because *dimm* is a prosecretory factor and enteroendocrine cells are secretory cells known to express a variety of peptide hormones (Nassel and Winther 2010). The pan-enteroendocrine marker Prospero (Pros) was used to visualize cells of the diffuse endocrine system along the length of the adult midgut. Pros^+^ staining is readily detected in individual endocrine cells, as are secretory neuropeptides, such as AstA, which is expressed in class I enteroendocrine cells of the posterior midgut (Figure 1, A and B′) (Beehler-Evans and Micchelli 2015). To test whether Dimm is normally present in wild-type adult enteroendocrine cells under baseline conditions, we examined the colocalization of anti-Dimm in Pros^+^ cells. Yet, Dimm protein was not detected in any cells of the adult midgut under our experimental conditions (Figure 1, C and C′), despite the fact that identical antiserum has been used to detect Dimm in other tissues (Hewes *et al.* 2003; Park *et al.* 2008).

We next examined Dimm protein distribution in the gut after exposure to the Gram-negative bacteria *Pe*. We first measured the survival of wild-type flies after exposure to *Pe ad libitum* at different culture densities (Figure 1G). We selected an intermediate value of OD 5 as the experimental exposure, because it was associated with high survival of wild-type flies during the first 48 hr after exposure. We estimate that OD 5 contained 2.3 × 10^9^ cells applied per vial (Table S4). In contrast to mock-treated controls, exposure to *Pe* at OD 5 was sufficient to induce anti-Dimm staining in Pros^+^ cells (Figure 1, D and D′). Upon induction, Dimm colocalized with nuclear DAPI, consistent with its characterized role as a transcription factor (Hewes *et al.* 2003; Park *et al.* 2011; Hadzic *et al.* 2015) and with markers of specific enteroendocrine cell subtypes (Figure 1D′ inset). When two different wild-type genotypes were used, Dimm induction was highly reproducible across independent trials (Figure 1H). Furthermore, adult midguts from *dimm* mutants did not display Dimm^+^ staining after exposure to *Pe*, despite the presence and normal density of Pros^+^ cells (Figure 1H and Figure S1). These results indicate that the immunoreactivity observed in wild-type enteroendocrine cells after bacterial challenge reflects changes in Dimm protein.

Exposure to *Pe* can lead to rapid production of new epithelial cells in the gut through a stem cell-mediated regenerative response (Jiang *et al.* 2009). It is therefore possible that the increase in Dimm signal reflects a part of the differentiation program of newly generated enteroendocrine cells. Alternatively, Dimm induction after *Pe* exposure could occur homeostatically in extant endocrine cells. To distinguish these possibilities, we examined Dimm immunostaining in the context of an adult midgut epithelium depleted of *escargot* (*esg*) expressing progenitor cells. We performed a conditional genetic ablation by initiating transgene expression of proapoptotic genes in adult progenitor cells (*UAS-rpr*, *UAS-hid*; *esgGal4*, *UAS-GFP*; *tub*Gal80*^TS^*). Ablation was confirmed by the absence of GFP^+^ progenitor cells and this manipulation alone was not associated with accumulation of Dimm (Figure S2). After exposure to *Pe*, midguts depleted of *esg*^+^ cells were capable of robust Dimm induction in Pros^+^ cells (Figure 1, E−F′). Taken together, we conclude that Dimm protein is normally low under baseline conditions, and is induced in mature enteroendocrine cells following exposure to *Pe*.

### Dimm induction in the diffuse endocrine system is transient and systemic

To further characterize Dimm induction in enteroendocrine cells, we performed a time course analysis ranging from 0 to 48 hr following initial *Pe* exposure. Four parameters were measured: (1) the percentage of individuals within an experimental group that displayed Dimm^+^ staining (percent Dimm^+^ midguts); (2) the percentage of Dimm^+^ endocrine cells per midgut (percent Dimm^+^, Pros^+^); (3) the anti-Dimm fluorescence intensity of individual enteroendocrine cells within a midgut; and (4) the fold induction of *dimm* mRNA measured by qPCR (Figure 2). Individuals displayed detectable Dimm^+^ cells between 3 and 24 hr after initial *Pe* exposure, with the percentage of positive midguts highest at 12 and 24 hr (Figure 2A). Dimm^+^ cells were not detectable in midguts from the 48-hr time point. Analysis of the percentage of Dimm^+^, Pros^+^ cells within midguts revealed that a majority of Pros^+^ cells become Dimm^+^ after *Pe* treatment (Figure 2, D and D′′). We independently examined data collected from the anterior, middle, and posterior midgut regions to identify any potential differences in either the timing or extent of Dimm induction. Regions did not significantly differ from each other within time points, with the exception of the 12-hr time point when the middle region was significantly higher than the posterior region (**ANOVA, F = 6.503). Consistent with this statistical result, we noted a general trend that the posterior midgut displayed lower percent Dimm^+^ values and that endocrine cells in the anterior region most proximal to the middle midgut were most consistently bright and of high percent induction. Fluorescence intensity of anti-Dimm staining in individual Pros^+^ cells was consistent with percent cell induction and showed higher values at the 12- and 24-hr time points (Figure 2B). Finally, to determine whether *dimm* is transcriptionally induced after *Pe* exposure, we compared *dimm* mRNA levels between mock- and *Pe*-treated flies by qPCR (Figure 2C). An increase in *dimm* transcript was detected at the 12- and 24-hr time points but not at 6- or 48-hr. Transcriptional induction was likewise reduced in *dimm* mutants (Figure S1). Taken together, we conclude that *dimm* is induced at the transcriptional and protein levels measured, and exhibit a similar time course showing highest values 12 and 24 hr after *Pe* exposure.

### Dimm induction in enteroendocrine cells is sensitive to low doses of *Pe*

We next asked whether Dimm induction varies with *Pe* dose. We exposed flies to *Pe* ranging from OD 0.001 to OD 10 and quantified the same set of parameters described previously (Figure 3). Although *Pe* doses of OD 0.001 and OD 1 were associated with very low lethality in wild-type flies (Figure 1G and Table S2), these doses were nevertheless sufficient to induce Dimm^+^ midguts at comparable frequency to OD 10 (Figure 3A). Similarly, *Pe* dose did not affect the percentage of Dimm^+^ endocrine cells assayed in different regions of the midgut (Figure 3, D−D′′). Thus, the percentage of Dimm^+^ enteroendocrine cells can reach high values at sublethal doses of *Pe*. Fluorescence intensity of anti-Dimm staining in individual Pros^+^ cells also reached high values at the relatively low dose of OD 0.001 (Figure 3B). Finally, examination of *dimm* mRNA levels by qPCR revealed that increasing *Pe* dose correlated with increased fold change in transcript (Figure 3C). Two transcripts previously characterized to be responsive to *Pe* infection, *unpaired 3* (*upd3)* and *Diptericin* (*Dpt)* (Vodovar *et al.* 2005; Jiang *et al.* 2009), showed a similar trend, suggesting that dose dependent transcriptional induction may be a general aspect of the response to *Pe* (Figure S3). Taken together, we conclude that independent measures of Dimm protein induction are sensitive to low doses of *Pe*, and that *dimm* transcript induction is dose dependent.

### Enteroendocrine cells express Dimm target genes in a *dimm*-dependent manner

The biosynthetic enzyme peptidylglycine alpha-monooxygenase (*Phm*), and *dcat-4*, which encodes a putative amino acid transporter, are direct transcriptional targets of Dimm in the embryonic central nervous system (Park *et al.* 2011). We therefore examined these targets in the diffuse endocrine system by immunostaining (Figure 4). After mock treatment, wild-type flies have detectable Phm in Pros^+^ cells despite a lack of Dimm staining in mock-treated flies (Figure 4A). Phm was also detectable in wild-type endocrine cells after *Pe* exposure (Figure 4B). However, Phm was not detectable in enteroendocrine cells of *dimm* mutants under mock- or *Pe*-infected conditions, despite the presence of Pros^+^ cells in mutant midguts (Figure 4, C and D and Figure S1, see the *Materials and Methods* section for description of genotype used). To test whether Phm is increased in midguts exposed to *Pe*, we quantified the percentage of Phm^+^, Pros^+^ cells after mock and *Pe* treatment. The percentage of Phm^+^, Pros^+^ cells was not significantly changed in response to *Pe* exposure (Figure 4E). We conclude that *dimm* is necessary for detection of Phm protein in adult midgut endocrine cells, and that Phm is detectable at similar levels in endocrine cells independent of *Pe* exposure.

Analysis of dCat-4 revealed detectable dCat-4 in a portion of wild-type Pros^+^ cells after mock and *Pe* treatment (Figure 4, F and G). In contrast to Phm, the percentage of dCat-4^+^, Pros^+^ cells was significantly increased as an effect of *Pe* (Figure 4J). *dimm* mutants produced detectable levels of dCat-4 protein; however, the percentage of dCat-4^+^ Pros^+^ cells did not increase as significantly as an effect of *Pe* in the absence of *dimm* (Figure 4, H, I, and J). We conclude that dCat-4 is a regulated target during the midgut response to *Pe*, and that *dimm* is not absolutely necessary for dCat-4 expression, but is necessary for dCat-4 induction in response to *Pe*. Taken together, we conclude that two previously identified direct transcriptional targets also show *dimm* dependent expression and induction in midgut endocrine cells.

### *dimm* is required for increased levels of AstA hormone after *Pe* infection

We hypothesized that Dimm induction in enteroendocrine cells could help support changes in peptide hormone levels after *Pe*. To test this possibility, we examined the protein AstA after mock and *Pe* treatment. AstA was detectable in both wild-type and *dimm* mutants under mock conditions (Figure 4, K and M). AstA also was detectable in both genotypes after *Pe* exposure (Figure 4, L and N). We noted a visual increase in the intensity of AstA staining in samples exposed to *Pe*, and therefore quantified the percentage of AstA^+^, Pros^+^ cells, as well as the fluorescence intensity of AstA antibody staining in individual cells (Figure 4, O and P). We observed that wild-type midguts significantly increased the percentage and levels of AstA after exposure to *Pe*. *dimm* mutants displayed a similar trend of AstA induction in response to *Pe*, however both the percentage of AstA^+^, Pros^+^ cells and the fluorescence intensity of individual enteroendocrine cells were less pronounced than that of wild-type flies. We conclude that AstA is a regulated peptide during the midgut response to *Pe* and that *dimm* is not absolutely necessary for AstA expression, but is necessary for normal AstA induction in response to *Pe* exposure. We note that in contrast to *Phm* and *dcat-4*, profiling studies in other tissues have not identified peptide hormones as direct targets (Park *et al.* 2011; Hadzic *et al.* 2015). Therefore, it remains unclear whether AstA is a direct transcriptional target in the gut or whether this regulation is indirect.

### *dimm* is a host factor that protects *Drosophila* against *Pe* infection

To test the functional requirement for *dimm*, we first examined the survival of *dimm* mutants after *Pe* challenge. Before treatment, *dimm* mutants displayed reductions in mass and midgut area (Figure S1, C and D). However, no significant effect on the density of Pros^+^ cells was detected (Figure S1E). After *Pe* exposure, survival of *dimm* mutants was significantly decreased compared with wild-type controls (Figure 5A and Table S3). Similar results were also observed following conditional expression of RNAi targeting *dimm* or *Phm* (Figure S4). To more specifically examine the involvement of *dimm* in the host immune response, we next compared wild-type and *dimm* mutant flies for their expression of a subset of antimicrobial peptides known to be involved in the innate immune response to *Pe* (Vodovar *et al.* 2005). The AMPs *Diptericin* (*Dpt)*, *drosocin* (*dro)*, and *Attacin A* (*AttA)* were highly induced in wild-type flies exposed to *Pe* (Figure 5B). However, *dimm* mutant flies displayed a significantly lower fold change in response to *Pe* for each AMP measured. Taken together, we conclude that *dimm* is a protective host factor that is necessary for the induction of antimicrobial peptides following infection by the Gram-negative pathogen *Pe*.

## Discussion

**Figure 6:**
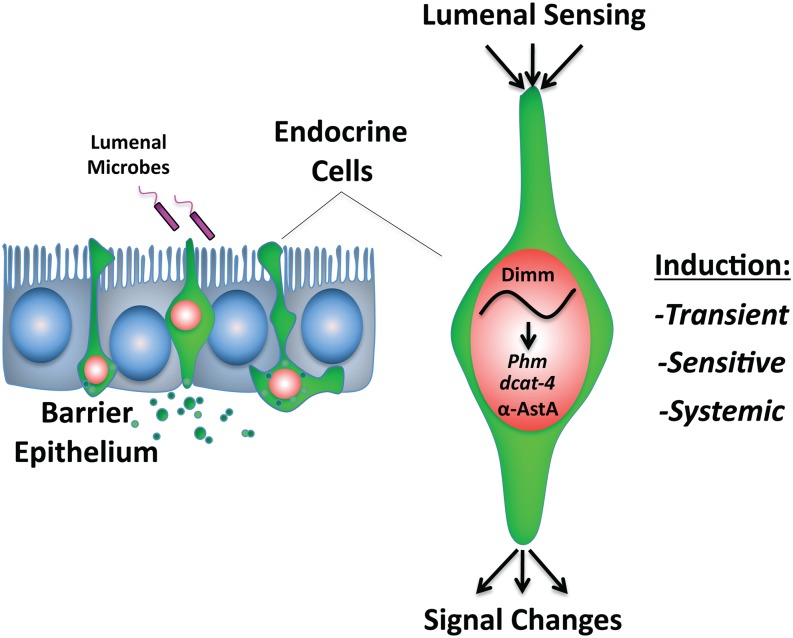
Model.

We used the *Drosophila* midgut to investigate how the diffuse endocrine system responds to pathogenic infection by a Gram-negative bacterium. Our studies establish an important new link between pathogenic infection and the coordinated induction of a prosecretory program in cells of the diffuse endocrine system. They also suggest a molecular model explaining how changes in the lumenal environment might result in a dynamically altered organismal physiology (Figure 6). Mature enteroendocrine cells respond to the pathogenic bacterium *Pe* with induction of *dimm*, a prosecretory basic helix-loop-helix transcription factor. This response shows a defined signature in several variables, including time, dose and region of enteroendocrine induction. In the diffuse endocrine system, *dimm* is necessary for normal levels of the important peptide hormone AstA and an enzyme necessary for the processing of peptide hormones more generally. Thus, Dimm may function to provide “gain” in the adaptive response of the diffuse endocrine system (Mills and Taghert 2012). Finally, we show that *dimm* is an essential host factor that protects the organism against pathogenic challenge and controls the induction of antimicrobial peptides. Future studies will determine the extent to which changes in the diffuse endocrine system directly regulate immune function or whether these processes occur in parallel.

## References

[bib1] Ahlman H. Nilsson, 2001 The gut as the largest endocrine organ in the body. Ann. Oncol. 12(Suppl 2)**:** S63–S68. 11762354 10.1093/annonc/12.suppl_2.s63

[bib2] Allan D. W. Park D. St Pierre S. E. Taghert P. H. Thor S., 2005 Regulators acting in combinatorial codes also act independently in single differentiating neurons. Neuron 45: 689–700. 15748845 10.1016/j.neuron.2005.01.026

[bib3] Beehler-Evans R. Micchelli C. A., 2015 Generation of enteroendocrine cell diversity in midgut stem cell lineages. Development 142: 654–664. 25670792 10.1242/dev.114959 PMC4325375

[bib4] Buchon N. Broderick N. A. Chakrabarti S. Lemaitre B., 2009 Invasive and indigenous microbiota impact intestinal stem cell activity through multiple pathways in *Drosophila*. Genes Dev. 23: 2333–2344. 19797770 10.1101/gad.1827009 PMC2758745

[bib5] Furness J. B. Rivera L. R. Cho H. J. Bravo D. M. Callaghan B., 2013 The gut as a sensory organ. Nat Rev Gastroenterol Hepatol 10: 729–740. 24061204 10.1038/nrgastro.2013.180

[bib6] Hadzic T. Park D. Abruzzi K. C. Yang L. Trigg J. S., 2015 Genome-wide features of neuroendocrine regulation in *Drosophila* by the basic helix-loop-helix transcription factor DIMMED. Nucleic Acids Res. 43: 2199–2215. 25634895 10.1093/nar/gku1377 PMC4344488

[bib7] Hamanaka Y. Park D. Yin P. Annangudi S. P. Edwards T. N., 2010 Transcriptional orchestration of the regulated secretory pathway in neurons by the bHLH protein DIMM. Curr. Biol. 20: 9–18. 20045330 10.1016/j.cub.2009.11.065 PMC2831871

[bib8] Helander H. F. Fandriks L., 2012 The enteroendocrine “letter cells”—time for a new nomenclature? Scand. J. Gastroenterol. 47: 3–12. 22126593 10.3109/00365521.2011.638391

[bib9] Hewes R. S. Park D. Gauthier S. A. Schaefer A. M. Taghert P. H., 2003 The bHLH protein Dimmed controls neuroendocrine cell differentiation in *Drosophila*. Development 130: 1771–1781. 12642483 10.1242/dev.00404

[bib10] Jiang H. Patel P. H. Kohlmaier A. Grenley M. O. McEwen D. G., 2009 Cytokine/Jak/Stat signaling mediates regeneration and homeostasis in the *Drosophila* midgut. Cell 137: 1343–1355. 19563763 10.1016/j.cell.2009.05.014 PMC2753793

[bib11] Kolhekar A. S. Roberts M. S. Jiang N. Johnson R. C. Mains R. E., 1997 Neuropeptide amidation in *Drosophila*: separate genes encode the two enzymes catalyzing amidation. J. Neurosci. 17: 1363–1376. 9006979 10.1523/JNEUROSCI.17-04-01363.1997 PMC6793724

[bib12] Lee W. C. Micchelli C. A., 2013 Development and characterization of a chemically defined food for *Drosophila*. PLoS One 8: e67308. 23844001 10.1371/journal.pone.0067308 PMC3699577

[bib13] Livak K. J. Schmittgen T. D., 2001 Analysis of relative gene expression data using real-time quantitative PCR and the 2(-Delta Delta C(T)). Methods 25: 402–408. 11846609 10.1006/meth.2001.1262

[bib14] Micchelli C. A., 2014 Whole-mount immunostaining of the adult *Drosophila* gastrointestinal tract. Methods 68: 273–279. 24680702 10.1016/j.ymeth.2014.03.022 PMC4430120

[bib15] Micchelli C. A. Perrimon N., 2006 Evidence that stem cells reside in the adult *Drosophila* midgut epithelium. Nature 439: 475–479. 16340959 10.1038/nature04371

[bib16] Mills J. C. Taghert P. H., 2012 Scaling factors: transcription factors regulating subcellular domains. BioEssays 34: 10–16. 22028036 10.1002/bies.201100089 PMC3692000

[bib17] Nassel D. R. Winther A. M., 2010 *Drosophila* neuropeptides in regulation of physiology and behavior. Prog. Neurobiol. 92: 42–104. 20447440 10.1016/j.pneurobio.2010.04.010

[bib18] Ohlstein B. Spradling A., 2006 The adult *Drosophila* posterior midgut is maintained by pluripotent stem cells. Nature 439: 470–474. 16340960 10.1038/nature04333

[bib19] Park D. Hadzic T. Yin P. Rusch J. Abruzzi K., 2011 Molecular organization of Drosophila neuroendocrine cells by Dimmed. Curr. Biol. 21: 1515–1524. 21885285 10.1016/j.cub.2011.08.015 PMC3184372

[bib20] Park D. Shafer O. T. Shepherd S. P. Suh H. Trigg J. S., 2008 The Drosophila basic helix-loop-helix protein DIMMED directly activates PHM, a gene encoding a neuropeptide-amidating enzyme. Mol. Cell. Biol. 28: 410–421. 17967878 10.1128/MCB.01104-07 PMC2223291

[bib21] Rehfeld J. F., 2004 A centenary of gastrointestinal endocrinology. Horm. Metab. Res. 36: 735–741. 15655701 10.1055/s-2004-826154

[bib22] Strand M. Micchelli C. A., 2011 Quiescent gastric stem cells maintain the adult *Drosophila* stomach. Proc. Natl. Acad. Sci. USA 108: 17696–17701. 21984734 10.1073/pnas.1109794108 PMC3203805

[bib23] Veenstra J. A., 2009 Peptidergic paracrine and endocrine cells in the midgut of the fruit fly maggot. Cell Tissue Res. 336: 309–323. 19319573 10.1007/s00441-009-0769-y

[bib24] Veenstra J. A., 2011 Neuropeptide evolution: neurohormones and neuropeptides predicted from the genomes of *Capitella teleta* and *Helobdella robusta*. Gen. Comp. Endocrinol. 171: 160–175. 21241702 10.1016/j.ygcen.2011.01.005

[bib25] Veenstra J. A. Ida T., 2014 More Drosophila enteroendocrine peptides: Orcokinin B and the CCHamides 1 and 2. Cell Tissue Res. 357: 607–621. 24850274 10.1007/s00441-014-1880-2

[bib26] Vodovar N. Vinals M. Liehl P. Basset A. Degrouard J., 2005 *Drosophila* host defense after oral infection by an entomopathogenic *Pseudomonas s*pecies. Proc. Natl. Acad. Sci. USA 102: 11414–11419. 16061818 10.1073/pnas.0502240102 PMC1183552

